# Dried Blood Spot Technique-Based Liquid Chromatography-Tandem Mass Spectrometry Method as a Simple Alternative for Benznidazole Pharmacokinetic Assessment

**DOI:** 10.1128/AAC.00845-18

**Published:** 2018-11-26

**Authors:** Danilo César Galindo Bedor, Noely Camila Tavares Cavalcanti Bedor, José Wellithom Viturino da Silva, Giovana Damasceno Sousa, Davi Pereira de Santana, Facundo Garcia-Bournissen, Jaime Altcheh, Bethania Blum, Fabiana Alves, Isabela Ribeiro

**Affiliations:** a Núcleo de Desenvolvimento Farmacêutico e Cosmético/Departamento de Ciências Farmacêuticas, Universidade Federal de Pernambuco, Recife, Pernambuco, Brazil; b B&S Inovação em Desenvolvimento e Análise de Produtos Farmacêuticos, Recife, Pernambuco, Brazil; c Parasitology and Chagas Service, Buenos Aires Children’s Hospital Ricardo Gutierrez, Buenos Aires, Argentina; d Drugs for Neglected Diseases *Initiative* (DND*i*), Geneva, Switzerland

**Keywords:** benznidazole, dried blood spots, LC-MS/MS, pharmacokinetics

## Abstract

Chagas disease (CD) is recognized as one of the major neglected global tropical diseases. Benznidazole (BNZ) is the drug of choice for the treatment of adults, young infants, and newborns with CD. However, the pharmacokinetics (PK) of BNZ have been poorly evaluated in all age groups, with consequent gaps in knowledge about PK-pharmacodynamic relationships in CD. The purpose of this study was to develop and validate a bioanalytical method to quantify BNZ levels in small-volume whole-blood samples collected as dried blood spots (DBS). The analysis was performed using high-performance liquid chromatography-positive electrospray tandem mass spectrometry. PK evaluation in healthy male volunteers was conducted to verify the correlation between DBS and plasma BNZ concentrations. The calibration curve was linear from 50 to 20,000 ng · ml^−1^. Intra- and interday precision and bias values were less than 14.87% (*n* = 9) and 9.81% (*n* = 27), respectively. The recovery rates ranged from 94 to 100% with no matrix effect. There was no hematocrit level effect in a range of 20 to 70%. The PK results obtained from DBS and plasma were comparable (*r*^2^ = 0.8295) and equivalent to previously published information on BNZ. BNZ in DBS was stable at room temperature for more than one year. This article describes the first microsampling method for measuring BNZ levels in DBS that has the potential to facilitate broad implementation of PK in clinical trials involving adult and pediatric patients in remote areas and helps to address existing knowledge gaps in the treatment of CD.

## Abstract (precis)

Chagas disease (CD) is recognized as one of the major neglected global tropical diseases. Benznidazole (BNZ) is the drug of choice for the treatment of adults, young infants, and newborns with CD.

## INTRODUCTION

Chagas disease (CD) is recognized by the World Health Organization (WHO) as one of the major neglected global tropical diseases. CD remains a significant public health problem, with social and economic impacts in several countries in Latin America, where it is endemic, affecting about 6 million people (1). In addition, CD is increasing in developed countries where it is not endemic due to globalization and population flow (2).

Presently, there are only two drugs available with proven clinical efficacy against Trypanosoma cruzi: benznidazole (BNZ) and nifurtimox, launched in the early 1970s (3). BNZ is widely accepted as the drug of choice for acute and early chronic-phase Chagas disease due to the large body of evidence from adequate and well-controlled studies (4). Safety and tolerability issues (such as hypersensitivity reactions, allergic dermopathy, and painful peripheral neuropathy) (5–7), as well as the long treatment duration, have limited broader use of the current compounds, particularly in the adult population, but these treatments have been shown to be manageable when used at a recommended dose of 5 mg/kg of body weight/day (8).

After a long period with limited information about pharmacokinetic (PK) parameters for BNZ (8, 9), various studies in patients and healthy volunteers have now been published. In a recent systematic review and meta-analysis, PK parameters were reported with a high degree of consistency across nine studies (2). PK values in healthy volunteers were different for men and women after oral administration of 100-mg tablets (8). Pediatric population PK was assessed in one clinical study, which showed lower BZN concentrations than those reported in adults, as well as fewer adverse events (10). However, until now, there has been no published data on PK in neonates and children under 2 years of age (10).

PK parameters are assessed using bioanalytical methods for the quantification of drugs in a biological matrix obtained by sampling in a clinical setting. There are a few methods reported in the literature for BNZ quantification in different matrices (tissues, plasma, serum, urine, and milk), notably differential pulse polarography high-performance liquid chromatography (HPLC) with ultraviolet detection (4, 11, 12), ultrahigh-performance liquid chromatography (UHPLC) with ultraviolet detection (13), and liquid chromatography coupled with tandem mass spectrometry (LC-MS/MS) (5, 14).

CD is most commonly diagnosed in poor regions with limited laboratory infrastructure and a lack of basic technologies for local bioanalytical testing. A noninvasive sampling method, allowing easy collection, storage, and transport of stable samples, is an important tool for conducting clinical trials in these settings, in particular those involving pediatric patients (15).

Over recent years, many reports have described the use of dried blood spots (DBS) (i.e., blood samples dried on a collection card) for quantification of drugs (15–21). DBS samples are commonly obtained from a finger prick using an automatic lancet on a specimen collection card. DBS sample collection is much easier and involves smaller sampling volumes than performing a venipuncture, and with proper training patients may even collect the samples themselves at home (20). Microsampling is often the preferred technique for pediatric PK studies (21).

The objectives of this study were to develop a DBS-based microsampling method for the quantification of BNZ in human blood for pharmacokinetic studies and to establish a new tool for PK assessment in patients with CD.

## RESULTS

### Selectivity, carryover, and cross talk.

The method used demonstrated excellent chromatographic selectivity, with no endogenous or metabolite interference at the retention times for both BNZ and the internal standard (IS) in six different sources of whole blood (one from postprandial collection) and in the presence of local anesthetic drugs. No residual chromatographic peak after ULOQ (upper limit of quantification) injection was observed after carryover evaluation. Even with good resolution (2.4) between the BNZ and IS chromatographic peaks, cross talk was evaluated and no interference between multiple reaction monitoring (MRM) channels was observed. For all tests, the response for blank DBS samples was within the required 20% of the average response of the lower limit of quantification (LLOQ) and 5% of the IS peak area.

### ME.

The matrix effect (ME), recovery efficiency (REC), and process efficiency (PE) were consistent over the tested concentration ranges for analyte low quality control (LQC) (67%, 109%, and 73%, respectively) and high quality control (HQC) (58%, 111%, and 71%, respectively).

The recovery results according to the FDA guidelines were 109%, 98%, and 111% for LQC, medium quality control (MQC), and HQC, respectively. The means from the QC samples was 106% ± 7%. IS has a recovery of 111%. The matrix factor (MF) values according to European Medicines Agency (EMA) guidance were 0.67 and 0.58 for LQC and HQC, respectively. The values found for normalized matrix factor (NMF) according to Brazilian Health Surveillance Agency (ANVISA) guidance were 0.98 ± 0.07 for LQC and 1.10 ± 0.03 for HQC. The value between two quality control levels was 1.04 ± 0.08.

### Linearity and sensitivity.

A calibration curve was constructed using eleven concentrations covering a range (50 to 20,000 ng · ml^−1^) derived from the peak area ratio of BNZ to IS. Weighted-least-square (WLS) linear regression, determined using 1/*x^2^*, was used to obtain linearity over four orders of magnitude, with a mean determination coefficient of 0.9840 (*n* = 3) and accuracy of back-calculated results of between 94.93 and 106.47%, as shown in Table 1. The lowest concentration at which BNZ could be quantified with an acceptable accuracy and precision was 50 ng · ml^−1^ (LLOQ).

**TABLE 1:** Precision and accuracy of concentration levels of calibration curve

Spiked concn (ng · ml^−1^)	Determined concn (ng · ml^−1^) (mean ± SD) (*n* = 3)	Precision (% RSD)	Accuracy (% RE)
50	47.994 ± 6.883	14.34	−4.01
100	101.828 ± 4.798	4.71	1.83
400	413.372 ± 32.867	7.95	3.34
800	812.695 ± 92.314	11.36	1.59
1,500	1,517.816 ± 7.750	7.75	1.19
3,500	3,674.722 ± 268.634	7.31	4.99
5,000	4,905.165 ± 483.027	9.85	−1.90
8,000	8,517.975 ± 343.651	4.03	6.47
12,000	12,256.665 ± 412.010	3.36	2.14
16,000	15,189.454 ± 549.700	3.62	−5.07
20,000	19,343.436 ± 942.394	4.87	−3.28

### Precision and accuracy.

The results for intra-assay and interassay precision and accuracy for the quality control samples at concentration levels of 50, 150, 10,000, and 17,000 ng · ml^−1^ for BNZ are summarized in Table 2. The intra-assay precision relative standard deviation (RSD; in percent) varied from 0.77 to 11.26%, and accuracy (percent deviation from nominal concentration) was 0.65 to 14.87%. The interassay precision RSD for samples at the LLOQ was 7.84%, and the interassay accuracy was 7.97%.

**TABLE 2:** Intra-assay and interassay imprecision of BNZ measurements on DBS

Nominal concn (μg · ml^−1^)	Found mean concn (μg · ml^−1^)	SD (μg · ml^−1^)	RSD (%)	RE (%)
Intra-assay (*n* = 9)				
0.05				
Day 1	0.053	0.003	5.30	6.05
Day 2	0.053	0.002	4.00	6.67
Day 3	0.056	0.006	11.26	11.20
0.15				
Day 1	0.165	0.008	4.75	10.10
Day 2	0.157	0.013	8.42	4.46
Day 3	0.172	0.006	3.71	14.87
10.00				
Day 1	10.197	0.382	3.75	1.97
Day 2	10.099	0.386	3.82	0.99
Day 3	11.157	0.169	1.51	11.57
17.00				
Day 1	18.142	0.736	4.05	6.72
Day 2	16.890	0.525	3.11	−0.65
Day 3	17.832	0.138	0.77	4.89
Interassay (*n* = 27)				
0.05	0.054	0.004	7,84	7.97
0.15	0.165	0.011	6.92	9.81
10.00	10.485	0.579	5.53	4.84
17.00	17.621	0.747	4.24	3.65

### Stability evaluation.

The results indicated that BNZ was stable under experiment conditions. Table 3 describes the back calculation and comparison with the nominal value.

**TABLE 3:** BNZ stability in DBS

Stability test	Nominal concn (μg · ml^−1^)	Found mean concn (μg · ml^−1^)	SD (μg · ml^−1^)	RSD (%)	RE
Benchtop	0.15	0.149	0.006	4.28	−0.38
	17.00	17.530	303.911	1.73	3.12
Autosampler	0.15	0.147	0.023	15.64	2.83
	17.00	15.884	449.986	−2.28	−6.56
Storage^*a*^	0.15	0.143	0.008	6.14	−4.35
	17.00	15.423	292.278	1.90	−9.28
Storage^*b*^	0.15	0.153	0.016	10.23	2.22
	17.00	18.200	1.420	7.80	7.06
Storage^*c*^	0.15	0.159	0.012	7.42	6.06
	17.00	18.046	1.011	5.60	6.15

a Storage lasted for 466 days at temperatures between 23°C and 26°C.

b Storage lasted for 7 days at 40°C.

c Storage lasted for 3 days at 40°C.

### Dried blood spot issues.

**FIG 1:**
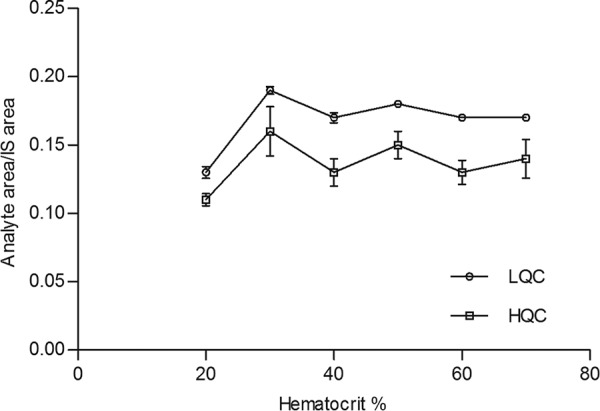
Hematocrit effect at 20%, 30%, 40%, 50%, 60%, and 70% in LQC and HQC samples.

The results show no significant influence of percent hematocrit on the precision and accuracy of the quantification of BZN (Fig. 1). The postcolumn infusion shows no matrix interference at the retention time of BNZ and IS at any hematocrit level (data not shown). The whole-blood-spot approach gives acceptable precision and accuracy results for all quality control levels, including LLOQ.

### Pharmacokinetic evaluation.

The results of PK parameters for DBS and plasma samples obtained from healthy volunteers are described in Table 4, including the DBS/plasma ratio for all parameters. The calculated area under the concentration-time curve from 0 h to time *t* (AUC_0-_*_t_*)/AUC_0-∞_ ratios were 0.79 and 0.92 for DBS and plasma, respectively. Hematocrit levels for volunteers were in the normal range (42.2, 45.3, and 43.5%).

**TABLE 4:** Pharmacokinetic parameters

PK parameter	Value for:		Ratio	*P* value^*a*^
	DBS (mean ± SD, *n* = 3)	Plasma (mean ± SD, *n* = 3)		
*C*_max_ (μg · ml^−1^)	2.070 ± 0.479	1.937 ± 0.225	1.07	0.4729
*T*_max_ (h)	2.83 ± 1.16	2.67 ± 1.44	1.06	0.4226
*k*_el_ (h^−1^)	0.041 ± 0.017	0.056 ± 0.008	0.74	0.2458
*t*_1/2_ (h)	19.38 ± 9.94	12.62 ± 1.80	1.54	0.3478
AUC_0-_*_*t*_* (μg · ml^−1^ · h^−1^)	26.623 ± 2.880	32.838 ± 5.116	0.81	0.0617
AUC_0–∞_ (μg · ml^−1^ · h^−1^)	33.691 ± 7.889	35.885 ± 6.499	0.94	0.7007

a *P* values were determined by two-tailed, paired *t* test, with 99% confidence intervals (GraphPad Prism, v. 5.03).

The calculated DBS concentration show a good degree of correlation with plasma concentrations, as described by *r* = 0.9108 (*P* < 0.0001; 95% confidence interval [CI], 0.08366 to 0.9521), but with significant negative bias for concentrations higher than 1,000 ng · ml^−1^.

**FIG 2:**
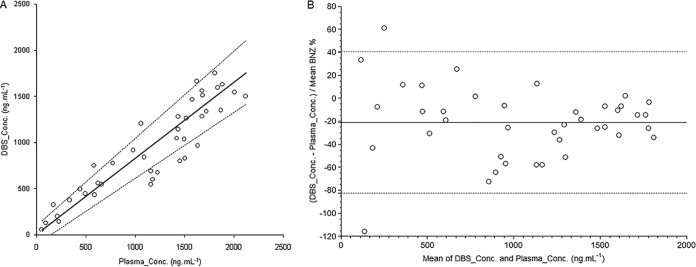
(A) Plot of BNZ concentration in DBS against BNZ concentration in plasma. The solid line is the line of true identity. (B) Bland-Altman plot for total plasma and DBS. The dotted lines indicate the 95% limits of agreement, and the solid line indicates the mean ratio bias.

The Passing-Bablok regression between paired BNZ-DBS and plasma concentration is plotted and Bland-Altman analysis are shown in Fig. 2. The slope of the regression line (Fig. 2A) was 0.84 (95% CI, 0.72 to 0.97), with an intercept of –8.72 (95% CI, −144.44 to 92.03). The Cusum test for linearity showed no significant deviation from linearity (*P* = 0.15). The Bland-Altman (Fig. 2B) plot shows a relative difference between DBS and plasma concentration of −20.9% ± 30%, with high bias for samples under 400 ng · ml^−1^.

The results of bioequivalence evaluation (46 volunteers finished the clinical study) showed maximum concentration of drug in serum (*C*_max_) of 2.53 and 2.48 µg · ml^−1^ and AUC_last_ of 50.22 and 48.93 h · µg · ml^−1^ for Radanil and LAFPE formulations of BNZ, respectively, and confidence intervals of 0.84 to 1.13 and 0.81 to 1.16 for *C*_max_ and AUC_last_, respectively (individual patient data are presented in the supplemental material). Formal comparison shows that these values meet the standard criteria for bioequivalence.

## DISCUSSION

To the best of our knowledge, this is the first report of a DBS–LC-electrospray ionization (ESI)-MS/MS bioanalytical method for determination of BNZ in whole-blood samples. A BNZ DBS assay was developed in combination with a fast, accurate, precise, and reproducible LC-ESI-MS/MS application. The assay was validated based on criteria described by FDA guidelines for bioanalytical method validation (22), taking into consideration the issues specific to DBS. In this method, the sampling procedure was simplified and requires significantly lower volumes of blood. In addition, the DBS can easily be stored and transported with a high degree of stability (23).

The mass spectrometer was set up to obtain the maximum signal without fragmentation in the ionization source and an MRM with the most sensitive and stable fragment to obtain a high signal/noise ratio (24). The chromatographic parameters were optimized to obtain separation (resolution, >1.5) between the analyte and IS with a good shape peak (tailing factor, <1.2), good run time (<5.0), and minimum matrix effect associated with the sample preparation technique.

The LC-MS/MS methods described in the literature give the results of an analytical run of 5.0 min with a large volume of biological matrix (2.5 ml urine) and a high volume of organic extraction solvent (2.5 ml dichloromethane), for a linear range from 10 to 50,000 ng · ml^−1^ (14), and an analytical run of 3.0 min with a small volume of biological matrix (50 µl serum) and a small volume of organic extraction solvent (250 µl of ethyl acetate), for a linear range from 100 to 3,000 ng · ml^−1^ (8). The method described in this article uses whole blood as the biological matrix at small volumes, a 5.0-min analytical run, and 1.7 ml dichloromethane in the extraction procedure, with a linear range of 50 to 20,000 ng · ml^−1^.

Differences were documented in the results of the matrix effect according to the EMA approach (with MF [see equation 4] at the same value of ME [see equation 1]) and the results according to the ANVISA approach (data from *C* samples are different for equation 3 [*C*/*A* × 100]). These findings are explained by the presence of the IS area ratio (see equation 5). Even with a nonstable isotope label (SIL), no influence of matrix for BNZ and IS was observed. The recovery of IS was 111% with no statistical difference, and recovery of BNZ was 106% (difference, −5.333 ± 3.767; *P* = 0.1803). According to the ANVISA approach, the percent coefficient of variation of the NMF should not be more than 15% in order to demonstrate the absence of any matrix effect. No limits for acceptance are described, but a range of 0.8 to 1.20 is commonly used (25). No significant signal suppression was observed. Accordingly, qualitative results after postextraction infusion demonstrates that the matrix effect is the same for the range of hematocrit levels studied for BNZ and IS analysis (20 to 70%).

Different hematocrit levels often lead to a diverse spread of blood onto the spot of the collection card, consequently the use of a center punch may result in the transfer of different blood volumes to sample preparation tubes. The current assay procedures involved the use of a whole-spot punch to avoid this effect (26); indeed, no influence of hematocrit levels was observed for the samples analyzed by this method, even with different levels of blood spread between the samples (27). The whole-spot approach with a fixed volume transferred by pipette can be considered a useful alternative that avoids hematocrit issues (15).

The method showed selectivity, linearity, precision, and accuracy, no matrix effect, no carryover, and stability of samples. In addition, potential DBS issues were avoided by the use of a fixed volume of blood and whole-spot analysis. Documented stability over a period of greater than 12 months at room temperature (between 20°C and ≤26°C) and stability over 7 days at 40°C with relative humidity of 53% is an important requirement for the application of this sampling method in remote regions where CD is endemic. The stability over the course of one week is important to guarantee sample integrity between blood collection and transport to a specialized laboratory and referral centers.

To the best of our knowledge, no similar study was reported in the literature to compare DBS sampling concentrations with plasma concentrations of BNZ. Although there was no significant deviation from linearity, we can see trends for underestimation of concentrations above 1,000 ng · ml^−1^ with the DBS method that contribute to high CI intervals for the intercept and for a high range of differences between sampling methods, as shown by Bland-Altman analysis, in addition to high variability, as samples under 400 ng · ml^−1^ showed results that were correlated as well as nonconcordant measures. For most clinical studies, this finding may have limited impact, as a single method is likely to be used. However, it is important to highlight this phenomenon when pooling or discussing data from different pharmacokinetic studies. Complementary evaluation of the relevance of this finding in patient samples also may be useful, as will be further analyses of DBS samples from venous and capillary collection.

Of note, a comparison of results from the 46 volunteers and those obtained by J. Raaflaub and W. H. Ziegler for the Radanil formulation of BNZ shows bioequivalence, with similar bioavailability after a single oral dose of 100-mg tablets. This is the only published information comparing the bioavailability of Brazilian BZN formulations to the older Roche products.

In resource-limited settings, with limited laboratory infrastructure and a lack of basic technologies for local bioanalytical testing, a noninvasive sampling method, allowing easy collection, storage, and less expensive transport of stable samples, is an important tool for conducting clinical trials, in particular those involving pediatric patients.

### Conclusions.

We have described a simple and rapid LC-MS/MS method for the quantification of BNZ with a DBS sampling technique, which showed selectivity and an acceptable level of precision and accuracy and adequate sensitivity and stability. This method was successfully applied to a clinical PK study of BNZ (100-mg tablets) in healthy male volunteers. A positive correlation with plasma concentration indicates that this method could be applied in adult and pediatric trials, thereby simplifying the conduct of PK studies in remote areas where CD is endemic and where only limited clinical and laboratory infrastructure is available. Additional studies at high temperature and high humidity settings would be useful to complement the current evaluation. Complementary evaluation of DBS in patients in the acute phase of infection and of the correlation of venous puncture and peripheral sampling method in diverse disease phases should be conducted.

## MATERIALS AND METHODS

### Chemicals and reagents.

One hundred-mg BNZ tablets and BNZ standard (99.47% purity; Nortec Química) were obtained from the Laboratório Farmacêutico do Estado de Pernambuco (LAFEPE; Recife, Brazil), and the internal standard (IS) citalopram hydrobromide was obtained from United States Standards (Rockville, MD).

All solvents used were HPLC grade. Ultrapure water was obtained from Milli-Q (Millipore Corporation, MA, USA). Formic acid (E. Merck, Darmstadt, Germany), acetonitrile (ACN), and isopropanol were supplied by J. T. Baker (Phillipsburg, NJ, USA), and dichloromethane (DCM) was supplied by Mallinckrodt Chemicals (Phillipsburg, NJ, USA).

### Preparation of stock and work solutions.

Two sets of stock solutions of BNZ were prepared in acetonitrile from two independent weighings at a target concentration of 1 mg · ml^−1^ free base. One stock solution was used to prepare calibration standards and the other to prepare QC samples. Stock solutions were further diluted with purified water to obtain separate working solutions in a range of 0.5 to 200 µg · ml^−1^ BNZ.

For the preparation of QC samples, separate working solutions were prepared with purified water at three concentrations: LLOQ, LQC, MQC, and HQC for BNZ (0.5, 1.5, 100, and 170 µg · ml^−1^, respectively).

Stock solutions of IS prepared in acetonitrile at a target of 10 µg · ml^−1^ free base were stored at −20°C until use. Stock solutions were further diluted with 1% formic acid in dichloromethane to a concentration of 3.3 µg · ml^−1^ (extract solution). Fresh extract solutions were prepared before each run.

### Preparation of calibration standards and QC samples in dried blood spots.

Calibration standards were freshly prepared from the whole-blood working solutions (human blood with EDTA as anticoagulant) to obtain BNZ in a calibration range of 50 (LLOQ) to 20,000 (ULOQ) ng · ml^−1^. Calibration standards were spotted on Whatman 903 filter paper cards by transferring 50 μl onto the card with a volumetric pipette. Thereafter, the blood spots were left to dry for a minimum of 3 h at room temperature. Quality control samples were prepared according to the calibration curve to obtain LLOQ (50 ng · ml^−1^), LQC (150 ng · ml^−1^), MQC (10,000 ng · ml^−1^), and HQC (17,000 ng · ml^−1^). Fresh calibration standards and QC samples were prepared before each run.

### Sample extraction.

Each DBS sample was punched into a vial with a standardized manual punch (13 mm) to obtain a whole-spot disk that was then extracted with 1,700 μl extraction solution (1% formic acid in dichloromethane) containing the internal standard, citalopram (3.3 µg · ml^−1^). Extraction was performed with a vortex for 30 min at room temperature (approximately 22°C). The supernatant (organic phase) was transferred to a clean tube and evaporated to dryness under a stream of nitrogen at 40°C in a nitrogen evaporator. The residue was reconstituted in 250 μl acetonitrile-water (1:1), and 150 μl was transferred to vials.

### LC-MS/MS method development.

The samples were measured using a Prominence analytical HPLC (Shimadzu, Kyoto, Japan) coupled to a Sciex 3200 QTRAP (SCIEX, Toronto, Canada) equipped with a Turbo IonSpray source and quadrupole analyzer. Infusion experiments were performed for multiple reaction monitoring (MRM) optimizations with a Harvard Apparatus 11 elite syringe pump (Hollston, MA) at a flow rate of 10 µl · min^−1^.

**FIG 3:**
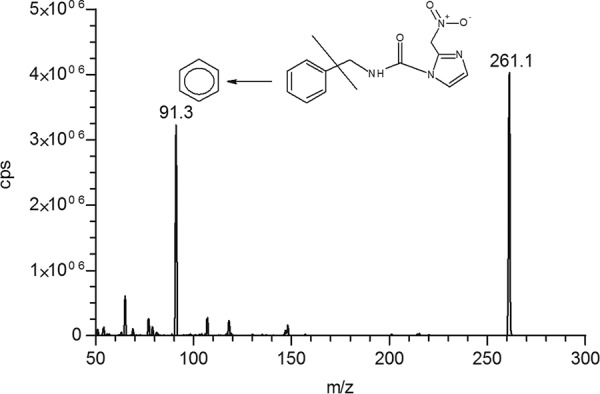
MS/MS product ion scans of BNZ, obtained by flow infusion analysis of 0.2 µg · ml^−1^.

An MRM method was prepared that included the most intense transitions for BNZ and the internal standard. Figure 3 shows BNZ positive ion electrospray mass spectra with the precursor and most intense product ions (*m/z* 261.1 > 91.3) and the mass transition, monitored in MRM mode, for IS. No fragmentation at the ionization source was observed by full scan monitoring.

The mass spectrometric parameters (cone voltage, collision energy, source temperature, desolvation gas, and multiplier detection) were optimized to obtain maximum sensitivity at unit resolution. The ESI was positive, capillary voltage was set to 5.5 kV, and heated turbo gas (air) was used with a flow rate of 45.0 liters · min^−1^ at 400°C. The following transitions were monitored in MRM mode: *m/z* 261.1 > 91.3 (quantifier) for BNZ and *m/z* 325.2 > 109.0 (quantifier) for IS. Optimal CXP (collision cell exit potential) was found to be 4 V for both BNZ and IS. The declustering potential (DP) applied was 41 V and 51 V for BNZ and IS, respectively. Collision energy (CE) was 41 V for BNZ and 37 V for IS.

Quantitation experiments were performed using a 20A LC system (Shimadzu Corporation, Kyoto, Japan) equipped with two analytical pumps (LC-20AD), a vacuum degasser (DGU-20A3), an autosampler (SIL-20AC HT), and a controller module (CBM 20A). The chromatographic column used was a Gemini NX (C_18_; 4.6 mm by 150 mm; 5 µm; Phenomenex, CA, USA), operating at 1.0 ml · min^−1^ for a total running time of 5 min. Isocratic elution was achieved using mobile phase A (water plus 0.1% formic acid) and mobile phase B (acetonitrile plus 0.1% formic acid) (43:57, vol/vol). The retention time was 2.9 min for BNZ and 1.3 min for IS with a total run time of 5 min. The peak asymmetry was 0.94 and 1.12 for BNZ and IS, respectively, and there was a resolution of 2.4 between peaks.

**FIG 4:**
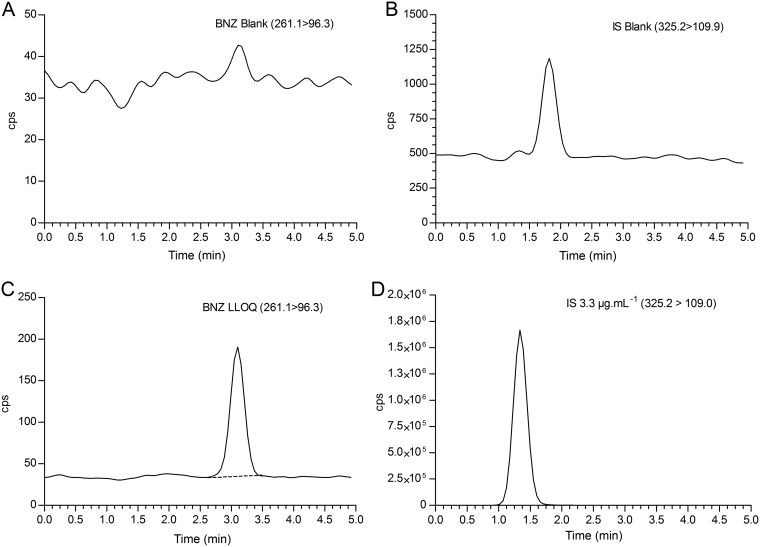
Extract ion chromatograms from blanks DBS of BNZ (A) and IS (B), as well as LLOQ of BNZ (C) and IS (D).

The eluent from the column was directed to the Turbo IonSpray probe without split ratio. Thirty microliters of the extracted sample was injected for the LC–MS/MS experiments, followed by a 0.8-ml strong needle wash consisting of 0.5% formic acid in acetonitrile–2-propanol–water (45:10:45, vol/vol/vol). Before analysis, samples were kept in an autosampler at 4°C. Reference chromatograms are shown in Fig. 4.

System control and data acquisition were performed with Analyst 1.5.2 software, including the Explore option (for chromatographic and spectral interpretation) and the Quantitate option (for quantitative information generation). Calibration curves were constructed with the Analyst Quantitation program with a linear WLS regression using 1/*x^x^*.

### Validation procedure.

Validation was carried out according to FDA guidelines for bioanalytical assays (22). There are particular issues for the validation of DBS methods, including the need for the evaluation of spot volume, punch location, and influence of hematocrit levels (28). In order to set up and validate the method, whole blood from healthy adult donors was spiked with BNZ at different concentrations. Fifty µl of each spiked blood sample was spotted on Whatman 903 filter paper and used for the calibration curve.

### Selectivity, carryover, and cross talk.

To evaluate the selectivity of the method, blank human whole-blood samples from five donors (human blood with EDTA as an anticoagulant) and from one postprandial donor were transferred to DBS and analyzed with a running time of 15 min (3-fold, as described above) for determining whether blood components interfere with the detection of the compounds of interest. For the evaluation of the interference between prilocaine and lidocaine (drugs commonly used as local anesthetics for sample collection in pediatric clinical trials), blank human blood samples were spiked with these compounds at a concentration of 1,000 ng · ml^−1^.

Carryover effect was evaluated by analysis of blank samples before and after injection of extracted samples at 17,000 ng · ml^−1^ and compared to an LLOQ response.

Cross talk interference was also evaluated by injection of nonextracted solutions (matrix free) of BNZ (17,000 ng · ml^−1^) and IS (2,000 ng · ml^−1^) separately.

### Matrix effect.

The recovery was assessed according to the guidance for industry bioanalytical method validation (18). The BNZ and IS recoveries were compared by unpaired *t* test (95% CI) (GraphPad Prism, version 5.00 for Windows; GraphPad Software, San Diego, CA USA, www.graphpad.com). For calculation of extraction efficiency, equation 3 was applied at three concentrations (LQC, MQC, and HQC).

In addition, the matrix effect (ME), recovery efficiency (REC), and process efficiency (PE) were also evaluated at LQC and HQC concentrations according to the following equations (29):

(1) ME(%)= B/A×100 

(2) REC(%)= C/B×100

(3) PE(%)= C/A×100

*A* is defined as the peak area of neat quality control samples prepared in acetonitrile, *B* is the DBS blank extracted and then spiked with BNZ in acetonitrile, and *C* is DBS spiked with BNZ and extracted.

To evaluate the influence of the matrix in bioanalytical response, two additional approaches were used. The matrix factor (MF) was obtained for two concentrations (LQC and HQC), according to European guidelines (30), by calculating the ratio of the peak area in the presence of matrix (*B*) to the peak area in absence of matrix (*A*) per the following equation:

(4) MF= B/A

Per guidelines from the Brazilian Health Surveillance Agency (ANVISA), the normalized matrix factor (NMF) was obtained for two concentrations (LQC and HQC), as described by the following equation (31):

(5) $$\text{NMF}=\frac{\text{BNZpeak area (}B\text{)}}{\text{ISpeak area (}B\text{)}}/\frac{\text{BNZpeak area (}A\text{)}}{\text{ISpeak area (}A\text{)}}$$

### Linearity, precision, and accuracy.

Human blood spiked with aliquots of a stock solution of BNZ to obtain blank samples, zero sample (blank plus IS), and 1 nonzero concentration, 50, 100, 400, 800, 1,500, 3,500, 5,000, 8,000, 12,000, 16,000, or 20,000 ng · ml^−1^, was spotted onto filter paper in order to prepare three different calibration curves. The linearity was assessed using the WLS method. The acceptance criteria for each back-calculated standard concentration was ±15% deviation from the nominal value, except at LLOQ, which was set at ±20%.

For precision (relative standard deviation) and accuracy (relative error [RE]) studies, four quality control samples (50, 150, 10,000, and 17,000 ng · ml^−1^) were prepared with nine replicates each and then analyzed on the same day (for intra-assay precision and accuracy studies) and on three different days (interassay precision and accuracy studies).

### Stability study.

For stability studies, quality controls were used at two concentration levels, LQC and HQC. The samples were processed, along with a fresh standard curve, and concentrations were determined. The percentage of degradation was obtained by comparing the back calculation of samples to the nominal value.

Benchtop stability was investigated to ensure that BNZ remained stable in DBS samples at room temperature for a period sufficient to cover the sample preparation procedure. DBS samples were left at room temperature (23°C) for 25 h. The samples were then processed and analyzed.

Due to the occasional need for delayed injection or reinjection of extracted samples, the stability of BNZ in the final solution was also evaluated in the autosampler at 4°C. A group of QC samples of BNZ was extracted, loaded onto the autosampler, and kept for 25 h before injection.

The following day, the samples were stored in sealed aluminum bags with three desiccant packages at four temperatures.

For evaluation of storage stability, the samples were prepared and air dried at 25°C for 3 h and then stored in sealed aluminum bags with two desiccant packages at room temperature (between 20°C and ≤26°C) for a period of 466 days. In addition, the samples were prepared and air dried at 40°C for 3 h and also stored in sealed aluminum bags with two desiccant packages at 40°C for 3 and 7 days. The humidity during the experiments was 53%.

### DBS issues.

The whole-blood-spot approach was used for the extraction procedure to avoid any interference of volume, to maximize the MS/MS signal, and to decrease the influence of hematocrit (26).

An evaluation of hematocrit on the DBS card was carried out. Human blood with EDTA as an anticoagulant was used to prepare samples at 20%, 30%, 40%, 50%, 60%, and 70% hematocrit. After centrifugation at 1,300 relative centrifugal force for 20 min, an appropriate volume of serum was added or removed to adjust the hematocrit (27).

The analyte/IS peak area ratio was compared with the response of DBS QCs at a hematocrit level of 45%, which was used for all validation parameter assessments.

The postcolumn infusion experiment was performed with injection of blank samples at each hematocrit level. The MS/MS response was monitored to verify the influence of different matrices on the retention time of analyte and IS.

### Pharmacokinetic evaluation and sample collection.

Forty-eight healthy volunteers from Brazil (24 men and 24 women) aged 18 to 45 years were included in this study based on their medical history, physical examination, 12-lead electrocardiography, and laboratory tests (hematology, blood biochemistry, hepatic function, and urinalysis) carried out before the study and at its conclusion. Inclusion criteria were weight within 15% of the ideal body weight, absence of heart, kidney, neurological, or metabolic diseases, and no history of drug hypersensitivity. Exclusion criteria were abnormal findings on physical examination, electrocardiography, or laboratory tests, ongoing pharmacological treatment, and history of alcohol or drug abuse. All subjects signed the consent form approved by the Ethics Committee of the Federal University of Pernambuco (CAAE; 35626814.0.0000.5208).

The study was an open-label, randomized, two-period, two-treatment, two-sequence, 2-by-2 crossover trial, balanced investigation of a single oral dose of BNZ (100-mg tablet) from Laboratório Farmacêutico da Marinha (Rio de Janeiro, Brazil) and the reference product (from LAFEPE) to evaluate the relative oral bioavailability of these formulations. Subjects were randomly assigned to one of two groups.

The treatments were administered in the morning with 200 ml water after a 10-h fasting period. No food was allowed for 3.25 h after ingestion of the dose. Subjects were provided with standard meals: lunch, a snack, and supper at 3.25 h, 8 h, and 12 h, respectively, after drug administration. The volunteers did not ingest any food or drink containing caffeine or xanthine during the trial.

Venous blood samples (5 ml) were collected before dosing and at 0.5, 1.0, 1.5, 2.0, 2.5, 3.0, 3.5, 4.0, 4.5, 5.0, 6.0, 8.0, 12.0, 23.0, and 47.0 h after dosing.

From these 48 volunteers, three were selected to obtain plasma and DBS samples. Before centrifugation, samples were spotted on Whatman 903 filter paper cards by transferring 50 μl blood onto the card with a volumetric pipette, and the tubes were then submitted to centrifugation for 5 min at 3,000 × *g*. Plasma samples were stored in polypropylene cryogenic tubes at −80°C until analysis. DBS samples were allowed to air dry for at least 3 h before being stored.

An evaluation of the relationship between BNZ blood and plasma concentrations was carried out by Passing-Bablok regression analysis of 40 (approximately 13 from each healthy volunteer) paired samples of DBS and plasma from the same sample collection, also applying the Bland-Altman approach (32). The correlation between sampling methods was described using Pearson correlation coefficient (*r*). All analyses used MedCalc statistical software, version 18.5 (MedCalc Software bvba, Ostend, Belgium, http://www.medcalc.org).

The PK parameters for BNZ determined for the plasma and blood matrices were *C*_max_, time to *C*_max_ (*T*_max_), AUC_0–_*_t_*, AUC_0–∞_, *k*_el_ (elimination rate constant), and *t*_1/2_ (elimination half-life). These were calculated by noncompartmental analysis using Phenix, version 7.0 (Pharsight Corporation, Sunnyvale, CA, USA). The *C*_max_ and *T*_max_ were obtained directly from the concentration-time curve. The AUC_0–_*_t_* was estimated by integration using the log-linear trapezoidal rule from time zero to the last measurable concentration at time *t. k*_el_ was calculated through the application of a log-linear regression analysis to at least the last three quantifiable concentrations of BNZ. *t*_1/2_ was calculated as 0.693/*k*_el_, and AUC_0–∞_ was calculated as AUC_0–_*_t_* + *C_t_*/*k*_el_, where *C_t_* is the last measurable BNZ concentration.

Additionally, a comparison between PK parameters (bioequivalence) from the Brazilian reference drug and published data for the Radanil formulation of BNZ (8) was carried out using Phenix, version 7.0 (Pharsight Corporation, Sunnyvale, CA, USA). Predefined bioequivalence parameters were consistent with FDA guidelines (33).

## Supplementary Material

Supplemental file 1
