# Supplementary material for: Dried Blood Spot Technique-Based Liquid Chromatography-Tandem Mass Spectrometry Method as a Simple Alternative for Benznidazole Pharmacokinetic Assessment
Source: Antimicrob Agents Chemother. 2018 Nov 26;62(12):e00845-18. doi: 10.1128/AAC.00845-18 (PMC6256767; doi:10.1128/AAC.00845-18)
Supplement: Supplemental file 1 [file zac012187671s1.pdf]

<u>Supplemental information</u> -

Individual Benznidazole (BNZ) Concentrations versus Time on Day 1 following single dose administration LAFEPE Benznidazole 100 mg tablets

Nominal_Time	BNZ Concentration (ng/mL)											
(hr)	ID #1	ID #2	ID #3	ID #4	ID #5	ID #6	ID #7	ID #8	ID #9	ID #10	ID #11	ID #12
0:00	0.00	0.00	0.00	0.00	0.00	0.00	0.00	0.00	0.00	0.00	0.00	0.00
0:30	2886.86	252.07	498.65	101.12	682.25	235.56	769.28	223.42	0.00	113.72	432.07	196.69
1:00	2600.91	735.97	738.49	239.93	1208.36	591.62	2008.03	335.67	0.00	264.50	1208.86	401.37
1:30	2312.87	1079.37	1118.22	398.79	1760.24	979.76	1672.96	624.21	59.03	219.02	1063.21	287.45
2:00	2492.45	1146.60	1753.41	627.42	2304.53	1511.09	1837.81	590.69	940.68	382.69	1529.49	424.25
2:30	2433.90	1603.29	2660.36	658.57	1550.27	1489.49	1885.33	1060.11	1106.80	545.88	1670.34	905.85
3:00	2102.78	1522.76	1900.09	1294.62	2753.15	1700.68	1857.23	1577.04	1327.62	731.05	939.07	915.40
3:30	2334.70	1768.93	1925.16	2353.37	1737.04	2543.95	1808.33	1685.49	1675.65	1803.85	1265.55	2071.57
4:00	2212.29	1603.96	1821.74	1689.35	2753.55	2461.75	1862.63	1517.19	1628.98	1762.02	1552.27	1669.07
4:30	2067.02	1592.42	1845.94	1757.91	2556.46	2129.50	1676.49	1499.16	1921.30	1674.19	1421.86	1746.08
5:00	2020.32	1437.37	1770.43	1630.02	2067.27	1874.89	1716.48	1491.71	1787.55	1559.69	2088.69	1488.22
6:00	1798.53	1404.90	2521.94	1632.55	2235.16	1831.86	1591.04	1415.25	1472.75	1552.33	1307.07	1829.32
8:00	1831.78	1393.66	1605.69	1582.56	2105.45	1953.17	1438.01	1223.07	2013.65	1410.95	1156.51	1362.07
12:00	1380.72	1036.58	1516.95	1272.74	1583.71	1616.84	1161.36	974.78	1766.48	1167.64	670.97	1063.81
23:00	775.86	639.51	882.82	769.02	680.77	1023.14	661.30	497.32	778.88	692.48	294.45	795.28
47:00	193.44	137.56	293.38	287.02	180.52	391.08	214.66	96.57	274.01	214.90	90.78	171.30
71:00	39.94	32.30	74.33	80.63	25.00	150.47	60.90	0.00	89.62	71.70	0.00	46.38

Note: Subjects ID#40 and ID#45 did not complete the study, thus are not included in the listing.

__Supplemental information__ -

Individual Benznidazole (BNZ) Concentrations versus Time on Day 1 following single dose administration LAFEPE Benznidazole 100 mg tablets

Nominal_Time	BNZ Concentration (ng/mL)											
(hr)	ID #13	ID #14	ID #15	ID #16	ID #17	ID #18	ID #19	ID #20	ID #21	ID #22	ID #23	ID #24
0:00	0.00	0.00	0.00	0.00	0.00	0.00	0.00	0.00	0.00	0.00	0.00	0.00
0:30	359.23	283.94	205.50	71.90	701.48	261.07	583.95	445.58	354.63	824.48	405.49	290.82
1:00	405.95	388.86	1512.43	283.25	1019.64	373.52	837.06	780.77	503.30	1286.88	2737.65	1502.38
1:30	568.95	413.01	2442.51	313.49	1444.44	1137.16	930.79	1077.90	1305.32	1408.91	3008.90	2329.80
2:00	1407.16	447.02	1972.23	304.53	2054.61	1729.24	1709.14	1084.20	1494.60	2430.50	2782.22	2466.74
2:30	1477.87	235.93	1978.23	321.93	2497.09	1810.61	2267.34	1213.72	2116.40	2694.51	2906.83	2442.70
3:00	1602.89	619.51	2047.51	348.48	2212.09	1583.16	2422.01	1214.60	2634.44	3034.02	2194.51	2467.77
3:30	1701.51	1623.91	1690.23	2259.27	2055.94	2191.35	3024.81	2332.91	1296.36	3004.33	2189.05	2479.24
4:00	1828.49	1628.03	1815.49	2559.03	2049.50	1995.31	2200.05	2008.26	1454.70	2977.54	3146.79	2223.34
4:30	1749.41	1627.84	1952.18	2265.35	1681.69	2066.46	2264.34	2066.11	1830.25	2874.98	2939.76	2239.54
5:00	1562.03	1616.70	2106.32	2480.25	2121.14	2037.81	2079.52	2124.88	1663.88	2728.82	2876.02	2186.70
6:00	1558.87	1578.65	1820.34	2504.11	2610.75	1785.89	1825.36	2016.14	1796.02	2662.82	2164.09	2233.22
8:00	1546.76	1398.95	1563.97	1994.96	1689.15	1636.76	1684.13	1894.12	1718.51	2265.08	1961.70	2070.05
12:00	1298.37	1176.54	1294.59	1428.19	1903.36	1153.96	1452.76	1440.08	735.54	1882.56	1769.10	1849.99
23:00	888.46	729.92	784.12	1145.93	978.83	517.21	792.06	815.14	448.74	1121.39	1376.88	855.84
47:00	327.64	244.72	245.83	261.47	203.02	143.15	194.89	216.85	101.64	327.30	303.05	284.84
71:00	111.08	63.37	67.71	44.23	59.17	27.31	39.40	49.65	0.00	74.50	85.40	132.27

Note: Subjects ID#40 and ID#45 did not complete the study, thus are not included in the listing.

Supplemental information -
Individual Benznidazole (BNZ) Concentrations versus Time on Day 1 following single dose administration LAFEPE Benznidazole 100 mg tablets

Nominal_Time	BNZ Concentration (ng/mL)											
(hr)	ID #25	ID #26	ID #27	ID #28	ID #29	ID #30	ID #31	ID #32	ID #33	ID #34	ID #35	ID #36
0:00	0.00	0.00	0.00	0.00	0.00	0.00	0.00	0.00	0.00	0.00	0.00	0.00
0:30	307.52	130.12	387.15	104.56	227.74	516.96	520.56	215.35	49.15	123.91	348.25	0.00
1:00	337.17	468.87	1006.89	404.41	393.33	852.54	1119.85	454.37	200.28	234.60	1075.68	106.81
1:30	3927.40	808.73	1285.83	671.40	491.18	1552.17	1237.92	570.43	466.76	1936.22	977.43	148.37
2:00	2209.48	677.79	1937.70	801.81	579.22	2736.09	2351.23	986.24	644.19	1866.40	1160.42	206.11
2:30	2457.83	1115.42	1499.21	1205.60	782.64	2957.20	2939.28	1295.62	619.53	1748.10	1385.33	1323.92
3:00	1940.03	1606.31	1480.45	2085.52	1215.51	3099.82	3159.27	1365.75	807.27	1702.29	1550.04	1380.64
3:30	2154.97	2692.35	2450.53	2295.51	2158.68	2710.72	2574.80	2789.87	1888.67	1766.13	2909.69	2442.02
4:00	2110.69	2228.22	1877.50	2272.98	2170.52	2715.93	2783.99	2974.61	1740.71	1912.93	2217.83	1281.67
4:30	1998.90	2440.43	1721.13	2317.69	2125.89	2625.33	2985.86	2818.52	1822.55	1734.86	1849.31	1450.00
5:00	1799.59	2351.90	2884.68	2179.02	2056.23	2720.35	2548.62	2705.74	1993.55	1760.95	2528.77	1489.63
6:00	1968.97	2474.06	3364.19	2132.87	1487.56	2548.66	2340.48	2571.01	1904.08	1929.89	2395.97	1480.36
8:00	1517.29	2187.94	2986.08	2010.99	1261.46	2539.13	2315.59	2341.85	1841.21	1556.05	1950.20	1404.19
12:00	1481.24	2063.89	2210.45	1524.29	1708.68	1940.09	1764.68	1987.39	1576.84	1207.72	1589.97	1249.36
23:00	920.93	1460.31	2068.30	936.87	985.90	1146.52	1056.45	1351.03	892.16	680.98	1100.30	735.37
47:00	212.15	611.53	384.83	293.63	145.02	300.83	280.89	433.25	210.83	175.94	423.15	194.09
71:00	45.32	213.54	113.99	89.16	34.01	70.55	69.75	162.62	47.45	42.91	89.61	39.03

Note: Subjects ID#40 and ID#45 did not complete the study, thus are not included in the listing.

Supplemental information -

Individual Benznidazole (BNZ) Concentrations versus Time on Day 1 following single dose administration LAFEPE Benznidazole 100 mg tablets

Nominal_Time	BNZ Concentration (ng/mL)									
(hr)	ID #37	ID #38	ID #39	ID #41	ID #42	ID #43	ID #44	ID #46	ID #47	ID #48
0:00	0.00	0.00	0.00	0.00	0.00	0.00	0.00	0.00	0.00	0.00
0:30	166.89	226.23	289.66	355.94	806.03	3383.76	170.96	408.00	394.01	89.41
1:00	477.56	507.50	755.58	615.00	1351.52	2601.28	732.14	670.16	2128.26	659.39
1:30	581.83	631.04	1817.92	2462.84	1630.91	3118.44	894.85	823.22	2102.77	1522.27
2:00	1573.69	553.40	2266.36	2391.35	1437.72	3009.95	1739.62	1013.22	2201.03	2133.15
2:30	1500.99	736.22	1919.30	821.33	2583.47	2382.61	1440.29	2007.23	2633.89	2490.72
3:00	2086.98	760.84	1895.41	2415.08	2972.29	3014.54	1723.22	2309.36	2479.33	2325.07
3:30	2326.27	2492.44	1932.45	2345.25	3151.48	3155.92	2047.28	2512.62	2102.47	2651.40
4:00	2149.52	3300.20	1550.66	2119.30	2759.19	2536.16	1895.82	2203.59	2578.91	2170.73
4:30	2185.48	2333.53	1584.80	2157.72	2705.39	2348.85	1707.26	2423.15	2295.30	2542.01
5:00	2195.46	2301.76	1703.45	1955.93	2828.51	2003.17	1718.36	2251.42	1946.10	2028.67
6:00	1964.53	1879.86	1507.77	1711.15	3410.93	2318.19	2070.34	2174.44	1713.02	1856.77
8:00	1943.47	1857.49	1319.71	1745.81	2573.14	2300.33	1991.88	1982.92	2366.45	1999.77
12:00	1494.54	1748.06	1161.63	1527.61	1910.06	1143.88	912.08	1676.22	1760.53	1608.23
23:00	903.11	1190.61	728.09	874.08	293.24	976.61	1217.30	967.54	699.58	735.45
47:00	292.13	399.40	216.25	322.50	1048.28	286.93	374.20	338.69	156.86	215.07
71:00	75.57	169.84	67.90	93.38	61.01	57.10	132.05	79.44	0.00	56.12

Note: Subjects ID#40 and ID#45 did not complete the study, thus are not included in the listing.
